# Characterizing a subtropical hypereutrophic lake: From physicochemical variables to shotgun metagenomic data

**DOI:** 10.3389/fmicb.2022.1037626

**Published:** 2022-12-02

**Authors:** Osiris Díaz-Torres, Ofelia Yadira Lugo-Melchor, José de Anda, Danielle A. Orozco-Nunnelly, Misael Sebastián Gradilla-Hernández, Carolina Senés-Guerrero

**Affiliations:** ^1^Centro de Investigacion y Asistencia en Tecnologia y Diseño del Estado de Jalisco, A.C., Unidad de Servicios Analiticos y Metrologicos, Guadalajara, Mexico; ^2^Departamento de Tecnologia Ambiental, Centro de Investigación y Asistencia en Tecnologia y Diseño del Estado de Jalisco, A.C, .Zapopan, Mexico; ^3^Department of Biology, Valparaiso University, Valparaiso, IN, United States; ^4^Tecnologico de Monterrey, Escuela de Ingenieria y Ciencias, Zapopan, Mexico

**Keywords:** subtropical lake, hypereutrophic lake, physicochemical parameters, shotgun metagenomic sequencing, pathogenic bacteria, biogeochemical cycling

## Abstract

Lake Cajititlán is a subtropical and endorheic lake, which is heavily impacted by nutrient pollution. Agricultural runoff and poorly treated wastewater have entered this reservoir at alarming rates during past rainy seasons, causing the cultural eutrophication of this body of water and resulting in several massive fish kill events. In this study, shotgun metagenomic sequencing was used to examine the taxonomic and functional structure of microbial communities in Lake Cajititlán during the rainy season. Several water quality features and their interactions with microbial communities were also assessed to identify the major factors affecting the water quality and biota, specifically fish species. According to current water quality regulations, most of the physicochemical variables analyzed (dissolved oxygen, pH, Secchi disk, NH_4_^+^, NO_3_^−^, blue-green algae, total phosphorus, and chlorophyll-*a*) were outside of the permissible limits. *Planktothrix agardhii* and *Microcystis aeruginosa* were the most abundant phytoplankton species, and the dominant bacterial genera were *Pseudomonas*, *Streptomyces*, and *Flavobacterium*, with *Pseudomonas fluorescens*, *Stenotrophomonas maltophilia*, and *Aeromonas veronii* representing the most abundant bacterial species. All of these microorganisms have been reported to be potentially harmful to fish, and the latter three (*P. fluorescens*, *S. maltophilia*, *A. veronii*) also contain genes associated with pathogenicity in fish mortality (*fur*, *lux*S, *aer*, *act*, *aha*, *exu*, *lip*, *ser*). Genetic evidence from the microbial communities analyzed herein reveals that anthropogenic sources of nutrients in the lake altered genes involved in nitrogen, phosphorus, sulfur, and carbon metabolism, mainly at the beginning of the rainy season. These findings suggest that abiotic factors influence the structure of the microbial communities, along with the major biogeochemical cycles of Lake Cajititlán, resulting in temporal variations and an excess of microorganisms that can thrive in high-nutrient and low-oxygen environments. After reviewing the literature, this appears to be the first study that focuses on characterizing the water quality of a subtropical hypereutrophic lake through associations between physicochemical variables and shotgun metagenomic data. In addition, there are few studies that have coupled the metabolism of aquatic ecosystems with nutrient cycles.

## Introduction

Anthropogenic activities and processes negatively impact water sources around the world, degrading the health of these systems, along with the ecological services they support and the biodiversity they harbor (Dokulil et al., 2000; de Anda and Shear, 2001; Skoulikidis et al., 2006; Skoulikidis, 2009; Kagalou et al., 2012; Kalogianni et al., 2017; Abell et al., 2019; Katsuki et al., 2019; Yu et al., 2020; Díaz-Torres et al., 2021).

In freshwater environments, phytoplankton make up the autotrophic component of the planktonic community and are thus the base of the food web. Massive phytoplankton blooms impact aquatic habitats by blocking light penetration and depleting oxygen at night (Ssebiyonga et al., 2013). In addition, certain genera of cyanobacteria (*Microcystis*, *Anabaena*, *Planktothrix*, *Oscillatoria*, *Anabaenopsis*, *Nostoc*) can be toxic to fish because they produce microcystins, a class of peptide toxins that can cause cell damage and organismal death by inhibiting phosphatases (Fu et al., 2005). Furthermore, some of the bacterial species can cause serious disease in fish. For instance, *Aeromonas veronii* can affect freshwater fish, amphibians, birds, and mammals (D’Aloia et al., 1996; Ghenghesh et al., 1999; Zhang et al., 2019). Moreover, *Pseudomonas fluorescens* is the most common culprit of fish illness and is frequently associated with skin and fin diseases (Pękala-Safińska, 2018). There have likewise been reports of health disorders and mortality in freshwater fish caused by *Shewanella putrefaciens*, *Acinetobacter* spp., and *Stenotrophomonas maltophilia* (Pękala-Safińska, 2018).

Changes in lake water quality impact microbial communities and their metabolic activities, altering biogeochemical processes such as sulfur, nitrogen, phosphorus, and carbon metabolism (Hupfer and Lewandowski, 2008; Liu et al., 2018; Wang et al., 2018; Yao et al., 2018). However, there is still limited knowledge on the interactions between water quality and biogeochemical processes within tropical and subtropical lake ecosystems (Díaz-Torres et al., 2021, 2022). Therefore, it is crucial to understand how microbial metabolism drives biogeochemical processes in these types of lakes that have high levels of nutrient pollution (Newton et al., 2011; Graham et al., 2016). Microbial metagenomics has proven to be quite valuable in informing remediation strategies for deteriorated ecosystems (Bowker, 2007; Steven et al., 2012). This technique allows for a fuller understand of the microbial community and what drives its structure, knowledge which is necessary for informed decision making (Guisan et al., 2013). Most of the reported metagenomic studies in lakes have been performed using amplicon sequencing, which is the most widely used approach for characterizing microbial diversity (Sharpton, 2014; Parulekar et al., 2017; Zhang et al., 2018; Nakatsu et al., 2019; Oyewusi et al., 2020). Amplicon sequencing studies in bacteria and archaea usually focus on the small 16S rRNA subunit, which is a phylogenetically and taxonomically informative marker (Hassler et al., 2022). Alternatively, in shotgun metagenomic sequencing, the entire genome is divided into small fragments and individually sequenced. This produces DNA sequences (reads) that align with multiple genomic regions (contigs) (Quince et al., 2017). As a result, metagenomic data allows researchers to investigate the following two elements of a microbial community simultaneously: 1) which organisms are present (bacteria, viruses, eukaryotes, archaea), and 2) what can they do (Hamady and Knight, 2009).

Lake Cajititlán is a small endorheic, subtropical, shallow lake located in the municipality of Tlajomulco de Zúñiga in the Mexican state of Jalisco (Díaz-Torres et al., 2021). This lake is both a symbol of identity and an important source of income for the inhabitants of Tlajomulco and the surrounding area, as the main economic activities of the region (e.g., tourism and fishing) depend on it. However, Lake Cajititlán is currently at risk of severe degradation due to the construction of new housing developments, the overexploitation of aquifers, and because of local, municipal, and agricultural wastewater that is being directly discharged to the lake (Caro-Becerra et al., 2017). Agriculture is the main economic activity in the Lake’s basin. Nevertheless, due to the excessive use of fertilizers, agricultural activities are one of the primary sources of nutrient pollution, resulting in cultural eutrophication process that is exacerbated by the endorreic nature of this reservoir (de Anda et al., 2019). Furthermore, the water quality of this lake is also affected by the lack of tertiary treatments to remove nutrients from wastewater, which is discharged into the lake from three municipal wastewater treatment plants (WWTPs) (de Anda et al., 2019). As a result, the lake has become hypereutrophic, triggering phytoplankton blooms, which have caused several massive fish kills between 2013 and 2022. All of these fish kill events occurred either during or immediately following the rainy season (Gradilla-Hernández et al., 2018; de Anda et al., 2019) due to the high rates of dissolved oxygen (DO) consumption by primary consumers during the night cycle, resulting in oxygen depletion (anoxia/hypoxia) (Gradilla-Hernández et al., 2018, 2020). Understanding the structure and function of microbial communities in freshwater lakes with severe anthropogenic pollution problems, like Lake Cajititlán, would greatly benefit remediation plans. Therefore, the objective of the present study was to assess the associations between water quality and microbial communities within Lake Cajititlán by analyzing several physicochemical variables along with shotgun metagenomic data during the rainy season. After reviewing the literature, this appears to be the first study that focuses on understanding the water quality of a subtropical hypereutrophic lake through associations between physicochemical variables and the structure and function of microbial communities using shotgun metagenomic sequencing.

## Methodology

### Study area

Lake Cajititlán is a subtropical endorheic lake in the state of Jalisco, located about 25 kilometers from Guadalajara, Mexico’s second largest city (Limón-Macías et al., 1983; Caro-Becerra et al., 2017, p. 21). The storage volume is 70.89 hm^3^, the maximum depth is 5.4 m, and the surface area is 1,744 ha (de Anda et al., 2019). As reported by Gradilla-Hernández et al. (2019), the hot-dry season in the area lasts from February to May, the rainy season is from June to September, and the cold-dry season is from October to January. According to monthly historical trends (1998–2018) in precipitation for Lake Cajititlán, July receives the most precipitation (7.60 mm), followed by August (5.93 mm) and then September (5.93 mm) (5.47 mm) (CONAGUA, 2021). Annual historical behavior for the TN:TP ratio and the ecosystem-specific water quality index (ES-WQI) for Lake Cajititlán has also been reported, with the lowest ES-WQI values in July and the highest fluctuations in the TN:TP ratio during the rainy season (Díaz-Torres et al., 2021).

**Figure 1:**
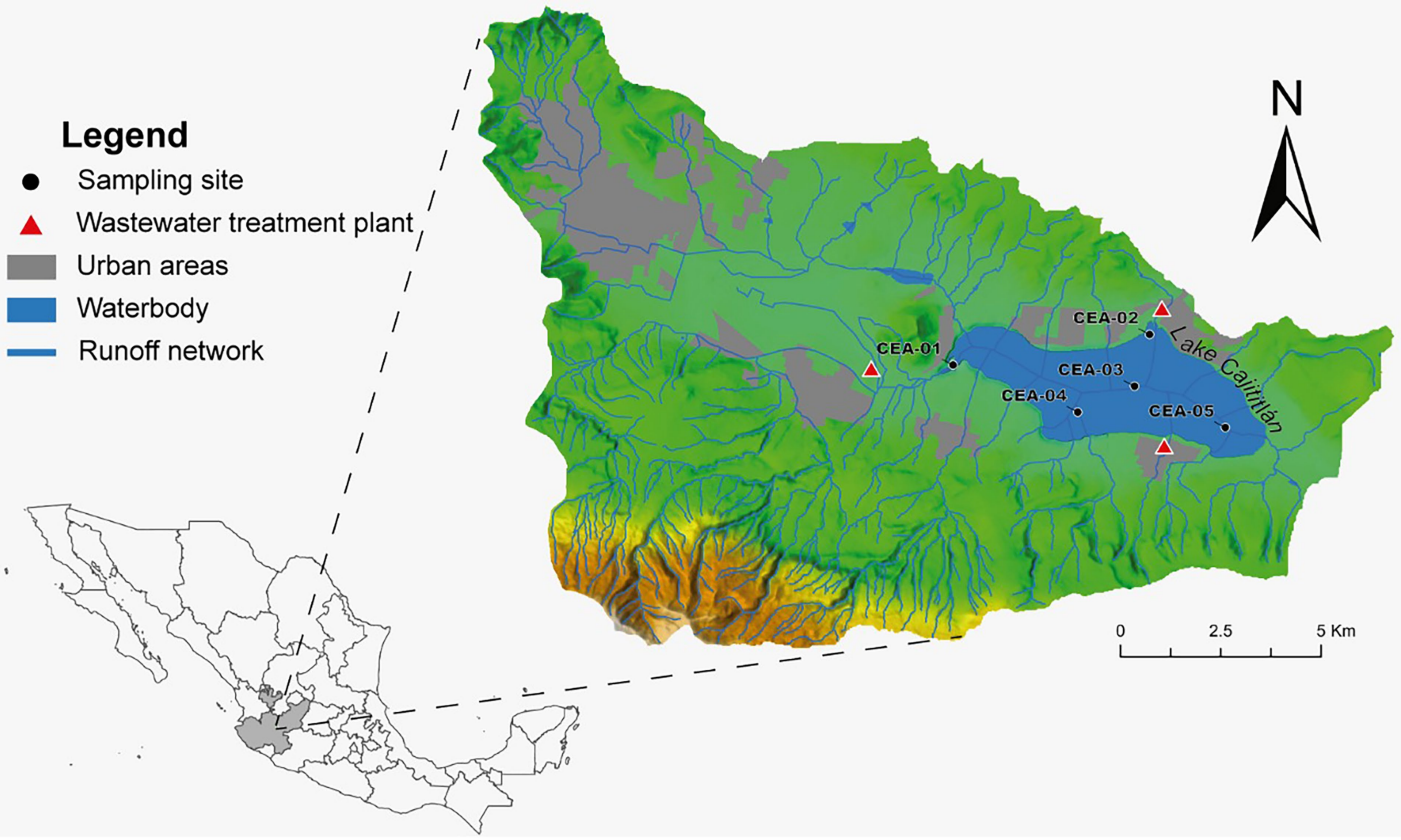
Locations of the sampling points in Lake Cajititlán.

Physicochemical data were measured *in situ* once a month from March to September 2018 at three depths (80 cm, intermediate depth, and interstitial) and at five sampling stations (CEA-1, CEA-2, CEA-3, CEA-4, and CEA-5; Figure 1). Dissolved oxygen (DO), electrical conductivity (EC), pH, water temperature (WT), nitrate (NO_3_^−^), ammonium (NH_4_^+^), turbidity, oxidation–reduction potential (ORP), phycocyanin-containing blue-green algae (BGA-PC), and chlorophyll-*a* were measured using two multiparameter probes (6,600 and 6,829 V2 YSI® at xylem brand, OH, United States) (YSI, 2009). Furthermore, during the rainy season (July–September) of 2018, water samples were taken once a month for shotgun metagenomic analysis, as these months have displayed the lowest water quality index values recorded in Lake Cajititlán and because the massive fish kill events were observed during these same months (Gradilla-Hernández et al., 2018; de Anda et al., 2019; Díaz-Torres et al., 2021). Water samples were collected using a Van Dorn water sampler at the same sites and depths that the physicochemical parameters were measured and were placed in disinfected 1 l plastic containers. Two duplicates of each sample (2 l each) were taken, for a total of 60 samples. Total nitrogen (TN) and total phosphorus (TP) measurements were provided by the State Water Commission (CEA by its Spanish abbreviation). These measurements were taken at a depth of 0.8 meters at the same sites and sampling months as the other physicochemical parameters.

### DNA extraction and metagenomic shotgun sequencing

Each water sample replicate was first filtered using a membrane with a 20–25 μM pore diameter. The filtrate was subsequently run through a second membrane with a 0.45 μM pore size. The FastDNA Spin Kit for Soil (MP Biomedicals, OH, United States) was then used to extract and purify the DNA from the samples, by adding 100 mg of each filter piece to a lysis matrix according to the manufacturer’s instructions. Quantification of DNA was conducted using a NanoDrop ND-1000 UV–Vis spectrophotometer (NanoDrop Technologies, Wilmington, DE). The DNA samples were then pooled by the depth of each site (CEA-1 to CEA-5) and sampling month (July to September; e.g., sample 1: CEA-1/July/80 cm and interstitial), to ultimately obtain 30 total samples (five samples per sampling month and its replica, July = 10, August = 10, and September = 10). The samples were then cleaned using the AM Pure XP kit (Beckman Coulter, IN, United States), and their DNA was quantified using the Qubit 2.0 fluorometer (Invitrogen, Carlsbad, CA, United States). Additionally, absorbance ratios at 260/280 and 260/230 nm were measured to analyze the purity of the samples. Finally, the samples were subjected to shotgun metagenomic sequencing on the Illumina® Novaseq6000 platform (Illumina, San Diego, CA, United States), yielding paired-end read lengths of 2 × 150 bp and 5 Gb of clean data per sample. Sequencing was carried out at the National Genomic Sequencing Laboratory Tec-BASE of Tecnologico de Monterrey.

### Bioinformatic analyses

OmicsBox 2.0.36 bioinformatics software was used to analyze and process the raw reads from the Lake Cajititlán samples (BioBam Bioinformatics, 2019). The reads were preprocessed using the Trimmomatic tool, which includes removing adapters and contaminant sequences, trimming bases, and filtering short, low-quality reads (Bolger et al., 2014). Subsequently, the samples were subjected to a quality control check using the FASTQ tool (Andrews, 2010). The final clean paired-end reads for each sample were individually subjected to metagenomic assembly using metaSPAdes, which is an assembly toolkit containing several assembly pipelines based on the de Bruijn graph (Nurk et al., 2017). The assemblages were then compared to the RefSeq v.2021_04 database using Kraken2 to obtain the microbial composition of Lake Cajititlán (Wood et al., 2019). Metagenomic gene prediction was performed using FragGeneScan, which is an application to find (fragmented) genes in short reads (Rho et al., 2010). The samples were then functionally annotated using the EggNOG mapper, which employs precomputed EggNOG-based orthology assignments (Huerta-Cepas et al., 2018). Finally, using the “Sample comparison GO chart” function, a comparative analysis of genetic ontology annotations was performed between the different samples of each month.

### Statistical analyses

All statistical analyses were carried out using the R (R version 4.1.2) software unless otherwise stated (R Core Team, 2021). All bar graphs and line plots were constructed using the ggplot2 package (Wickham, 2016). The heatmap was developed using the dist() function and the pheatmap package (Kolde, 2019). Mean values and standard deviations (SD) were calculated for each physicochemical parameter by sampling month with the mean() and sd() functions. One-way analyses of variance (ANOVA) (α = 0.05) were performed to test for temporal differences in water quality features. The mean physicochemical parameter values were compared to different national standards and international recommendations (Vollenweider and Kerekes, 1981, 1982; Salas, 2003; WHO, 2003; MRCCC, 2013; LFD, 2019; NOM-001, 2021).

A box plot was created to depict the relative read abundances of Lake Cajititlán’s microbial communities at the domain-level (archaea, bacteria, eukaryotes, and viruses). The relative read abundance of each of the 30 samples was calculated individually, and the results were plotted by sampling month. All boxplots were constructed to incorporate the results of one-way ANOVA (α = 0.05) and Tukey’s HSD tests, which were performed using the stats package’s TukeyHSD function (R Core Team, 2021). Phytoplankton species and bacterial genera were examined using bar graphs of relative read abundance over the sampling month. Bar charts were created using the scales packages. Taxa of phytoplankton species and bacterial genera that were found in proportions of less than 0.01%, were categorized as “others” (Wickham and Seidel, 2022).

Principal coordinate analysis (PCoA) was also performed to investigate differences in phytoplankton species and bacterial genera across the sampling months. The vegan package and the cmdscale function were used to calculate distances based on Bray-Curtis differences (Oksanen et al., 2022). Furthermore, a pairwise analysis of variance (ADONIS) was also conducted using the vegan package and the adonis2() function to test the significance of temporal changes in the composition of Lake Cajititlán’s bacterial and phytoplankton communities (Clarke, 1993; Anderson, 2001; Anderson et al., 2006). Additionally, a redundancy analysis (RDA) was performed to determine the relationship between these communities and the physicochemical parameters using the vegan and ggord packages (Beck, 2021; Oksanen et al., 2022).

A literature search was conducted to analyze which bacterial species have been reported as pathogenic to fish using different terms or keywords such as pathogenic bacteria, fish pathogens, fish pathogenic bacteria, fish bacterial disease, fish diseases, and fish pathogens, among others. This search was performed using PubMed, PubMed Central, ScienceDirect, JSTOR and Scopus databases, resulting in 22 articles relevant to the objective (Almodóvar et al., 2004; Ellermeier and Slauch, 2007; Geng et al., 2007; Swain et al., 2007; Ilardi et al., 2009; Wang et al., 2009; Geng et al.,2010a,b; Loch and Faisal, 2010; Troxell et al., 2010; Dang et al., 2011; Johnson et al., 2011; Humbert et al., 2013; Ngwa et al., 2014; Liu et al., 2015; Harke et al., 2016; Menanteau-Ledouble et al., 2016; Bukowska et al., 2017; Pękala-Safińska, 2018; Basri et al., 2020; Li et al., 2020; Shabana et al., 2022). These species were then compared with the taxonomic data obtained herein and were displayed using a relative read abundance on a chord diagram created with the circlize package (Gu et al., 2014). In addition, a heatmap was built using the virulence factors that play an important role in the development of diseases caused by the most abundant species by sampling month. These analyses were carried out to infer potential synergistic factors (i.e., bacterial diseases, changes in the physicochemical parameters of the water, harmful cyanobacteria, eutrophication, and anthropogenic activities) that could have triggered the massive fish kill events in Lake Cajititlán.

Genes involved in the metabolism of carbon, nitrogen, and sulfur, as well as in the phosphorus cycle were searched for in the KEGG database^1^, and the abundance of the group of genes by metabolic pathway was shown through bar charts of relative read abundance per sampling month using the phyloseq and vegan packages (Kanehisa et al., 2015; McMurdie and Holmes, 2013; Oksanen et al., 2022). Finally, the metabolic pathways that were statistically significant for each sampling month were thoroughly analyzed, and a graphic representation of the metabolic pathways, their stages, and the genes involved was created, followed by a Euclidean distance heatmap to visualize gene abundance per sampling month (Kolde, 2019).

## Results

### Water quality characteristics

All physicochemical parameters, except for TN and TP, showed significant temporal fluctuations when comparing the values reported for the seven sampling months (Table 1). Most of these parameters were outside the permissible limits. However, March (which is in the hot-dry season) showed the fewest parameters outside the limits. WT and EC were the only variables that were within acceptable limits for all the months analyzed. The lowest ORP values and highest turbidity values were observed in July, whereas the highest TN values were observed in June, the onset of the rainy season (Gradilla-Hernández et al., 2019). Compared with the limits established by the applicable Mexican regulation, the TP, DO, and pH were outside of the permissible limits for all months, except for the DO, which was found to be within these guidelines for March and June (Table 1).

**Table 1:** Water physicochemical features of Lake Cajititlán between March and September of 2018.

Parameter	Unit	Acceptable limit	March Mean ± SD	April Mean ± SD	May Mean ± SD	June Mean ± SD	July Mean ± SD	August Mean ± SD	September Mean ± SD
DO*	mg/L	5.00^1.1^	6.14 ± 3.22	3.96 ± 3.29	4.43 ± 4.53	6.59 ± 3.39	2.83 ± 0.61	3.25 ± 1.87	4.32 ± 1.77
pH*	–	6.50–8.50^1.1^ 6.9^2^	9.19 ± 0.07	8.70 ± 0.10	8.82 ± 0.11	9.66 ± 0.14	10 ± 0.05	9 ± 0.05	9.28 ± 0.19
WT*	°C	35.00^2^	20.77 ± 0.84	21.57 ± 1.23	23.72 ± 1.29	23.24 ± 0.66	24.16 ± 1.55	24.73 ± 1.12	24.35 ± 0.34
Secchi disk*	cm	<150.00^5^ Hypereutrophic	8.35 ± 3.30	8.45 ± 2.56	8.30 ± 0.60	10.06 ± 1.48	9 ± 1.14	21.8 ± 7.57	8.2 ± 8.09
ORP*	mV	–	188.35 ± 18.35	137.74 ± 40.14	120.47 ± 57.03	193.19 ± 30.11	47.90 ± 12.36	66.18 ± 19.30	101.78 ± 7.04
**NH**_4_^+^*	mg/L	0.06^1.1^	2.74 ± 0.07	2.83 ± 0.67	2.55 ± 0.72	3.02 ± 0.17	4.50 ± 0.38	4.08 ± 1.46	2.49 ± 0.92
**NO**_3_^−^ *	mg/L	0.04^1.2^	3.16 ± 0.23	4.59 ± 0.42	4.21 ± 0.12	3.10 ± 0.62	3.67 ± 0.34	2.67 ± 0.56	0.87 ± 0.24
BGA-PC*	cell/mL	100,000^4^	272,458 ± 3,752	272,446 ± 8,133	274,154 ± 9,548	278,447 ± 7,608	275,129 ± 5,551	167,662 ± 79,268	268,078 ± 3,996
Chlorophyll-a*	μg/L	>25.00^5^ Hypereutrophic	47.30 ± 8.40	45.30 ± 9.74	52.92 ± 11.29	40.53 ± 2.54	42.16 ± 5.20	42.83 ± 7.40	47.40 ± 9.36
EC*	mS/cm	0–1.5^3^	1.00 ± 0.01	1.07 ± 0.02	1.04 ± 0.01	1.11 ± 0.005	1.08 ± 0.003	1.21 ± 0.16	1.16 ± 0.12
**TN**	mg/L	–	11.33 ± 1.77	12.51 ± 3.75	14.21 ± 5.58	15.87 ± 4.67	11.46 ± 1.08	12.88 ± 2.52	9.85 ± 0.63
**TP**	mg/L	0.05^1.1^	1.58 ± 0.06	1.42 ± 0.13	1.64 ± 0.09	1.58 ± 0.06	1.51 ± 0.06	1.52 ± 0.24	1.41 ± 0.10
		>0.1^5^ Hypereutrophic							
Turbidity*	NTU	–	76.84 ± 2.17	88.11 ± 9.32	94.99 ± 10.55	106.89 ± 4.13	97.40 ± 5.87	42.17 ± 6.46	68.94 ± 7.13

The asterisk symbol in the parameters denotes statistical significance among months at a *p* < 0.05 level. Underlined values indicate that they exceed acceptable limits. Dissolved oxygen (DO), pH, water temperature (WT), Secchi disk, oxidation–reduction potential (ORP), ammonium (NH_4_^+^), nitrate (NO_3_^−^), blue-green algae (BGA-PC), chlorophyll-*a*, electrical conductivity (EC), total nitrogen (TN), total phosphorus (TP), and turbidity.^1.1^ Use 1. Aquatic life protection: freshwater, includes wetlands (LFD, 2019); ^1.2^ Use 2. Aquatic life protection: coastal waters and estuaries (LFD, 2019); ^2.^NOM-001, 2021; ^3.^MRCCC, 2013; ^4,^WHO, 2003; ^5^Vollenweider and Kerekes, 1981, 1982; Salas, 2003.

### Bioinformatic analysis

**Figure 2:**
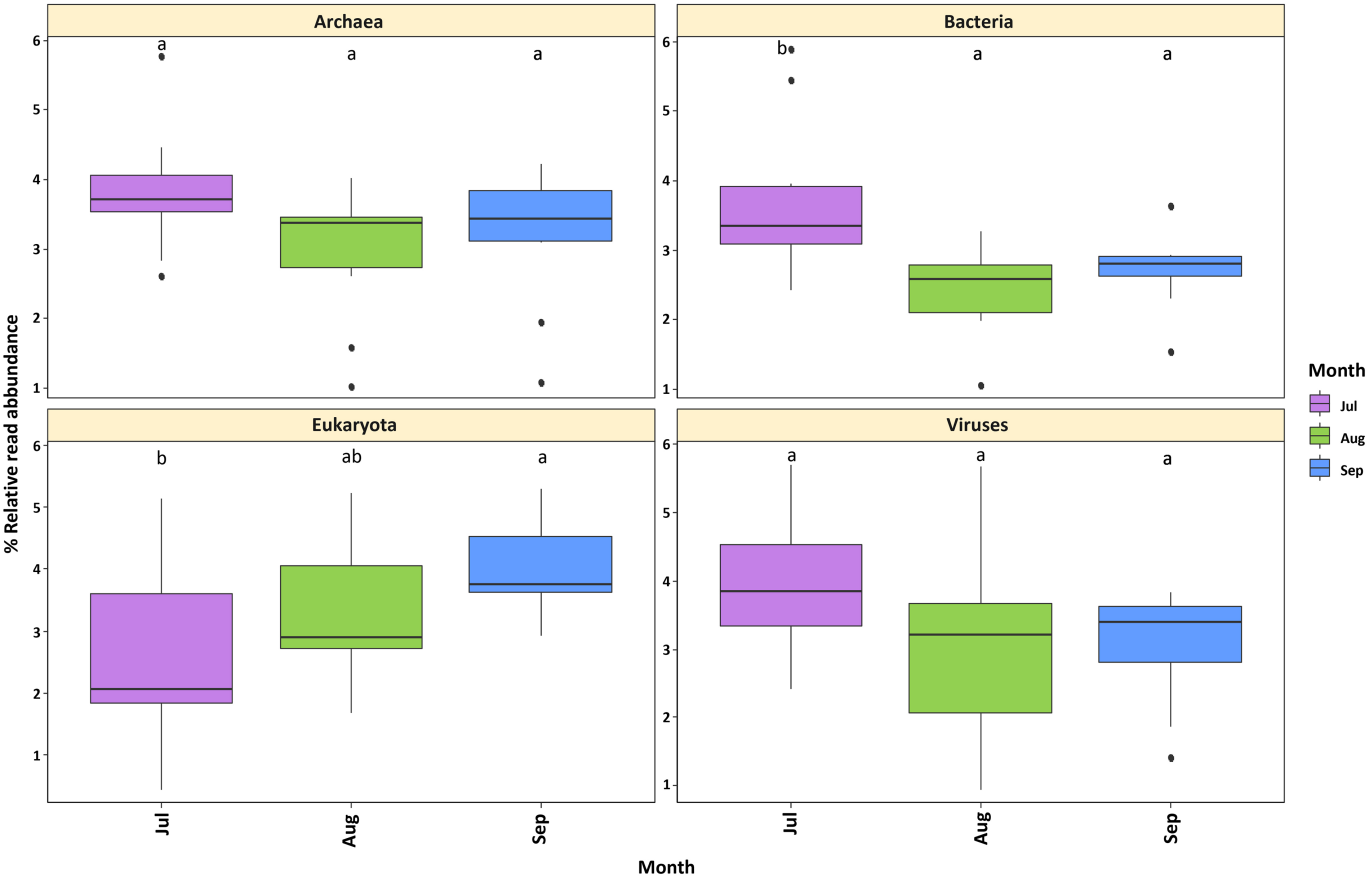
Relative read abundance of Lake Cajititlán’s microbial communities at the domain level by sampling month. **(A)** Archaea. **(B)** Bacteria. **(C)** Eukaryota. **(D)** Viruses. Relative read abundances were calculated separately for each domain.

The sequencing of all samples resulted in a total of 748,721,392 raw reads and 748,721,392 contigs (Supplementary Table S1). Kraken2 was used to compare the reads to the RefSeq v.2021_04 reference database, resulting in 29,544,581 total reads (49.87%) that were classified in one of the following taxonomic domains: bacteria (46.45%), eukaryotes (3.17%), archaea (0.27%) and viruses (0.07%) (Supplementary Table S1). Regarding EggNOG Mapper results, a predicted gene annotation of 12,491,565 (> 18%) was obtained (Supplementary Table S2). Classified reads per month ranged from 49.98 to 51.72% (Supplementary Table S2). Statistical analyses revealed no significant differences in the abundance of archaea and viruses per sampling month (Figure 2). However, temporal differences were detected in the bacteria and eukaryotes. Furthermore, an inverse trend was observed between these two domains in the first 2 months (July and August); while the relative read abundance of bacteria decreased, the abundance of eukaryotes increased (Figure 2).

### Temporal variations of the diversity and abundance of phytoplankton and bacterial communities

**Figure 3:**
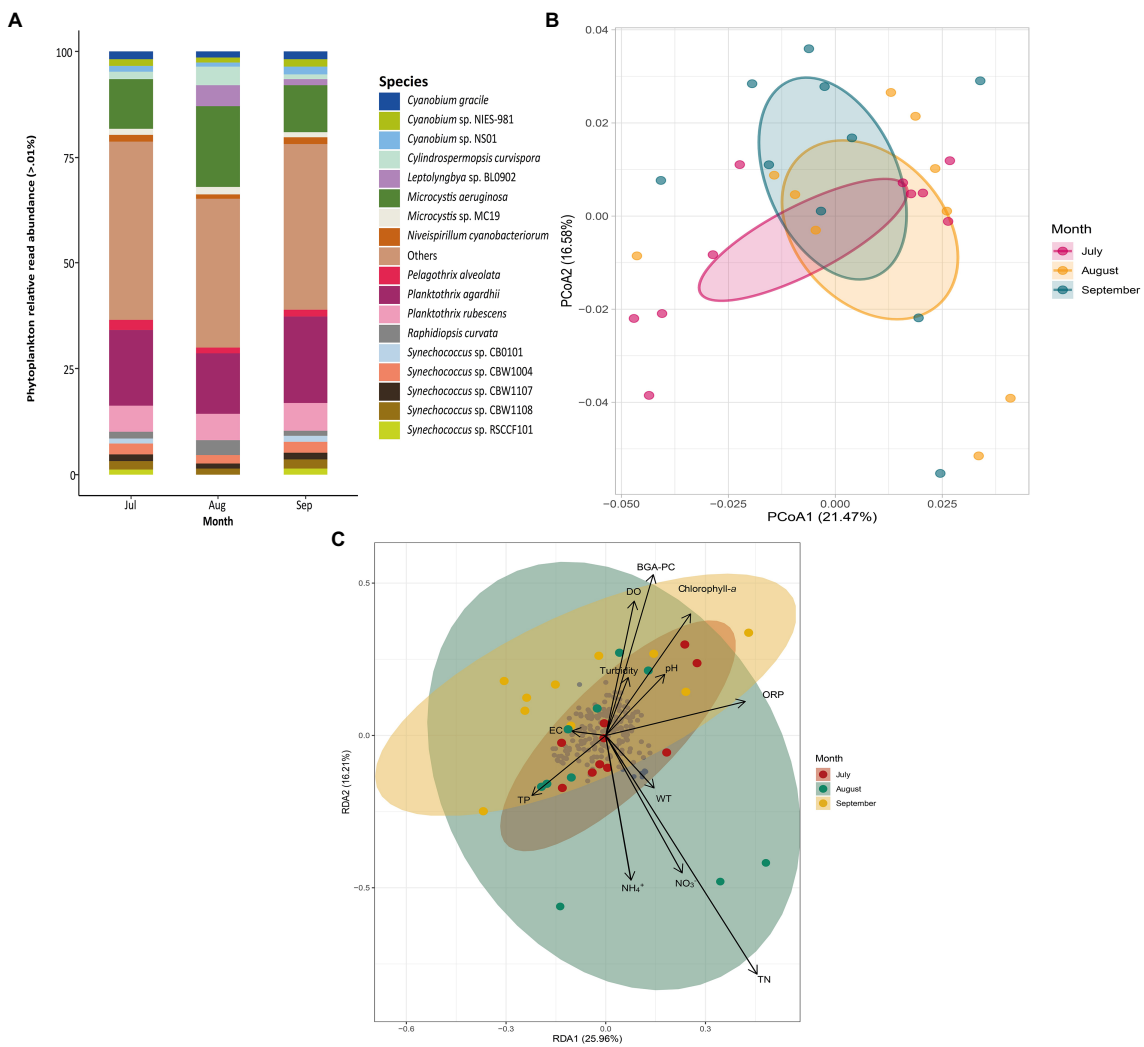
**(A)** Phytoplankton species relative read abundance by sampling month. **(B)** PCoA of phytoplankton communities by sampling month. **(C)** RDA of phytoplankton composition and physicochemical variables by sampling month. Dissolved oxygen (DO), pH, water temperature (WT), oxidation–reduction potential (ORP), ammonium (NH_4_^+^), nitrate (NO_3_^−^), blue-green algae (BGA-PC), and chlorophyll-*a* and electrical conductivity (EC) total nitrogen (TN), total phosphorus (TP), and Turbidity.

**Figure 4:**
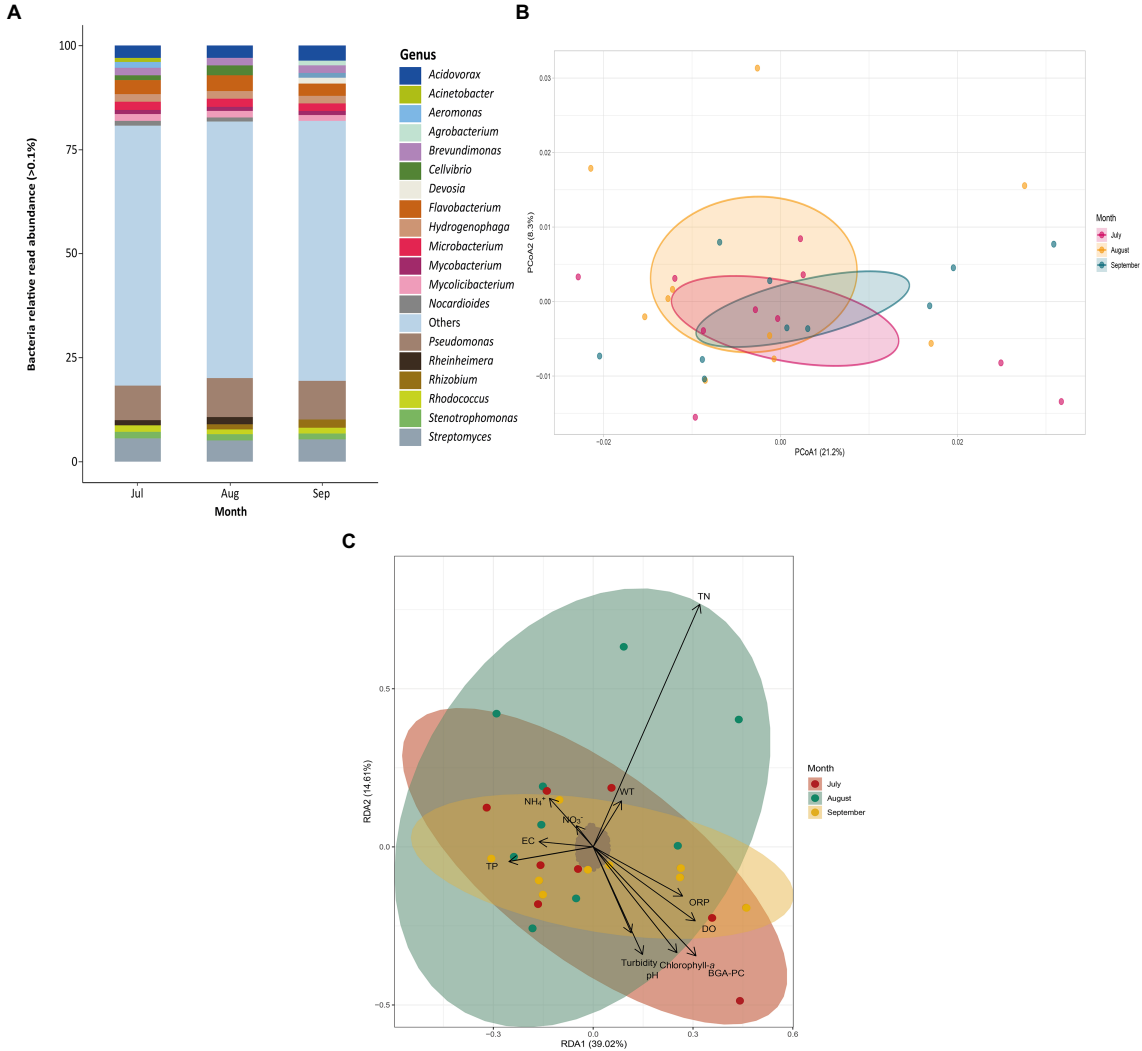
**(A)** Relative read abundance of bacteria genera by sampling month. **(B)** PCoA of bacterial communities by sampling month. **(C)** RDA of bacterial composition and physicochemical variables by sampling month. Dissolved oxygen (DO), pH, water temperature (WT), oxidation–reduction potential (ORP), ammonium (NH_4_^+^), nitrate (NO_3_^−^), blue-green algae (BGA-PC), and chlorophyll-a and electrical conductivity (EC) total nitrogen (TN), total phosphorus (TP), and Turbidity.

The diversity and abundance of phytoplankton (Figure 3) and bacterial communities (Figure 4) were assessed at different sampling months using relative read abundance bar plots. The temporal differences were examined using PCoA. RDAs were then used to evaluate the relationship between the presence and prevalence of these communities and the physicochemical variables of Lake Cajititlán. Within the phytoplankton communities, *Planktothrix agardhii* (17.70%) and *Microcystis aeruginosa* (14.00%) were consistently the most abundant species during all sampled months (Figure 3A). The *Pseudomonas* (8.95%), *Streptomyce*s (5.32%), and *Flavobacterium* (3.36%) bacterial genera were the most abundant in all sampled months (Figure 4A). Significant temporal variations were only detected in the composition of the phytoplankton (*p* < 0.05) and bacterial (p < 0.05) communities between July and September according to the ADONIS analysis (but not observed with the PCoA analysis) (Figure 3B, 4B; Supplementary Table S3, S4).

According to the RDA, the physicochemical factors that were most correlated with the taxonomic composition of the phytoplankton communities during July were TP and pH. Similarly, NH_4_^+^, NO_3_^−^, and TP were most correlated with the communities in August and EC and turbidity were most correlated with the taxonomic composition in September (Figure 3C). The first three redundancy components explained 55% of the variability (RDA1: 25%; RDA2: 17%; RDA3: 13%; Supplementary Table S5). The first component was positively correlated with ORP. The second component was negatively correlated with NH_4_^+^ and TN, but positively correlated with BGA-PC. Turbidity, ORP, pH, and NO3-were the most highly (positively) correlated parameters with the third component (Supplementary Table S6). Likewise, WT, NH_4_^+^, chlorophyll-*a* and BGA-PC were most correlated with the taxonomic composition of the bacterial communities in July. NH_4_^+^, TP, and TN were most correlated with the communities in August, whereas TP, ORP, and DO were more correlated with the taxonomic composition in September (Figure 4C). The first three components explained 61% of the variability (RDA1: 39%; RDA2: 14%; RDA3: 8%; Supplementary Table S7). DO, BGA-PC, and TN were most (positively) correlated with the first component. The second component was most correlated with TN (positive correlation), along with BGA-PC and chlorophyll-*a* (both with negative correlations). DO and TP were the most highly (positively) correlated with the third component (Supplementary Table S8).

Parameters with positive correlations may imply that microorganisms thrive in environments with higher values of these physicochemical factors. Conversely, negative correlations indicate that higher values of these parameters negatively influence the predominance of microbial populations during the study period (Liu et al., 2014).

### Bacterial species and their virulence factors reported as pathogenic for fish

**Figure 5:**
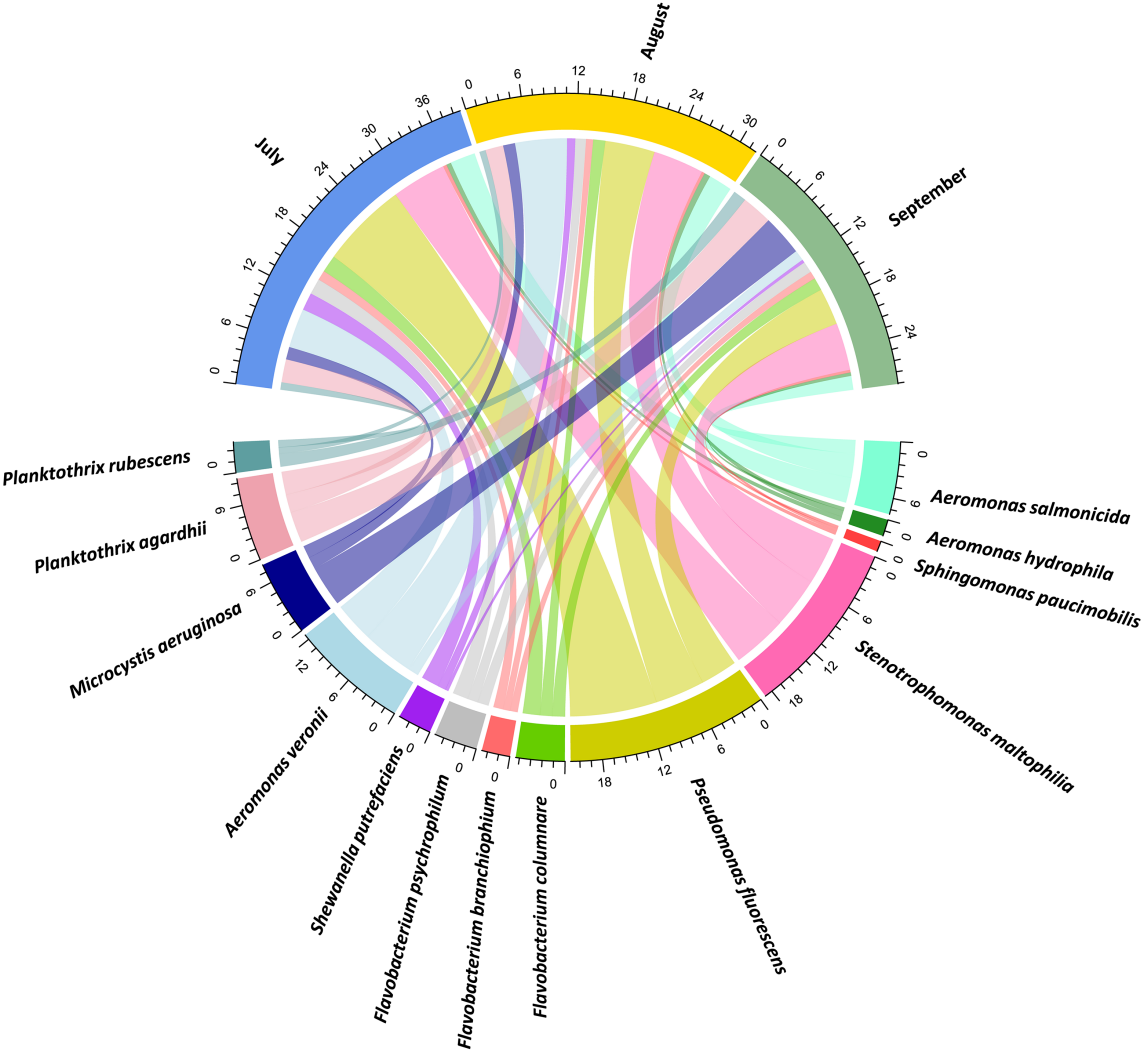
Chord diagram showing the distribution of bacterial species that have been reported as pathogenic for fish. The numbers around the circumference represent the percentage of relative abundance coverage of the bacterial species by sampling month in Lake Cajititlán.

A literature search was carried out to determine which bacterial species have been reported as fish pathogens in other freshwater bodies to compare with this study. *Pseudomonas fluorescens* (21.37%), *Stenotrophomonas maltophilia* (18.86%), and *Aeromonas veronii* (12.58%) were the most abundant species, as shown in in Figure 5. The monthly abundance of *P. fluorescens* (50.03%) was higher in July, and its abundance decreased throughout the sampling months. The relative abundance of *S. maltophilia* remained constant during the three-month investigation. The highest abundance of *A. veronii* was observed in August (50.73%), followed by July (38.74%) and September (10.53%). The remaining pathogenic bacteria in the chord diagram were found to be less abundant for all 3 months of study.

*P*. *fluorescens* is one of the main causes of septicemic diseases in freshwater fish, causing severe economic losses in aquaculture (Swain et al., 2007; Wang et al., 2009; Shabana et al., 2022). This species is usually associated with pathologies of the skin and fins of fish and has also been reported to cause sudden mortality in fish (Pękala-Safińska, 2018). *A*. *veronii* is a major pathogen causing sepsis and ulcer syndrome in freshwater fish (Li et al., 2020). *S*. *maltophilia* has been reported to cause infectious intussusception syndrome in these freshwater animals (Geng et al., 2007, 2010a,b). The virulence factors that play an essential role in the development of diseases caused by *P. fluorescens* and *A.* *veronii* in fish are depicted in a heatmap (Supplementary Figure S1). So far, no information has been found on virulence genes associated with *S. maltophilia* fish diseases. For *P. fluorescens* to be infectious to the host, the acquisition of iron is essential, which is related to the pathogenic *fur* gene (Ellermeier and Slauch, 2007; Troxell et al., 2010; Johnson et al., 2011; Liu et al., 2015). The highest relative abundance of the *fur* gene occurred in July, followed by September, while the fewest were observed in August (Supplementary Figure S1). The genes responsible for diseases (sepsis and ulcer syndrome) caused by *A. veronii* are as follows: *aer, act, aha, ser, exu, lip,* and *lux*S (Li et al., 2020). The heatmap shows a trend like that of the *fur* gene, with all virulence genes most abundant in July, followed by September, and then August. The *exu* (nuclease) gene was the most abundant gene, while *lux*S (quorum sensing controlled gene) was the least abundant (Li et al., 2020*;* Supplementary Figure S1*).*

### Influence of microbial communities on biogeochemical cycles

**Figure 6:**
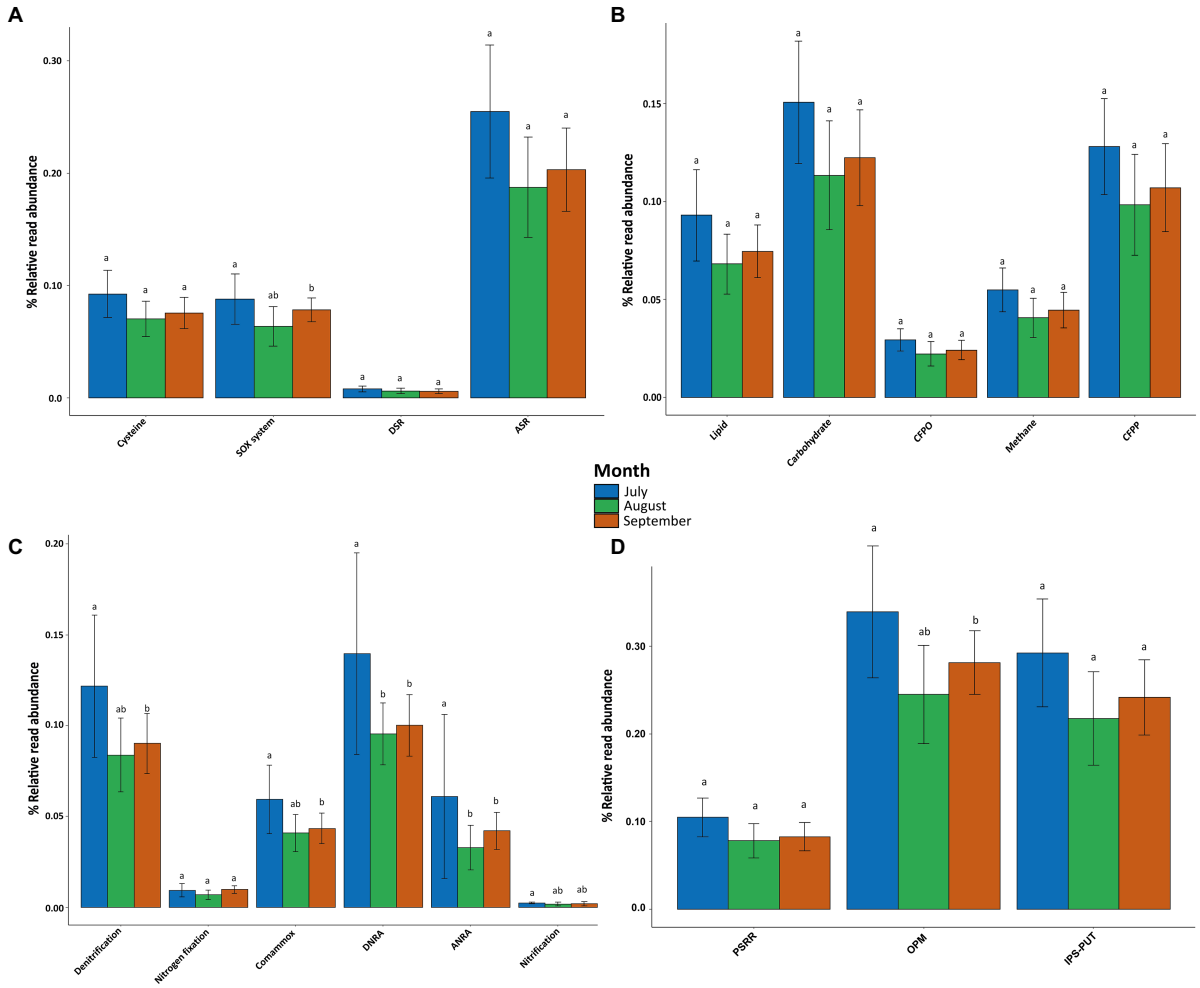
Relative read abundance of genes associated with major pathways in **(A)** Sulfur metabolism–ASR, assimilatory sulfate reduction; DSR, dissimilatory sulfate reduction; SOX system, sulfur oxidation system. **(B)** Central carbon metabolism–CFPP, carbon fixation pathways in prokaryotes; CFPO, carbon fixation in photosynthetic organisms. **(C)** Nitrogen metabolism–DNRA, dissimilatory nitrate reduction to ammonia; ANRA, assimilatory nitrate reduction to ammonia. **(D)** Phosphorus cycle–IPS-PUT, Inorganic P solubilization and P uptake and transport; OPM, organic P-mineralization; PSRR, P-starvation response regulation. Different letters indicate significant differences within a single pathway.

The relative read abundances of functional genes involved in the biogeochemical cycles for nitrogen, phosphorus, carbon, and sulfur were calculated based on annotated reads (Figure 6). In general, all of the biogeochemical cycle pathways were more abundant in July, then decreased in August, and slightly increased in September. The genes associated with sulfur metabolism only displayed significant differences between July and September (value of *p* = 0.0352; Figure 6A) for the sulfur oxidation system (SOX system). No significant differences were identified regarding central carbon metabolism (Figure 6B). The genes associated with nitrogen metabolism showed significant differences for all of the following pathways: the denitrification pathway (July vs. September: value of *p* = 0.0169), the comammox pathway (July vs. September: value of *p* = 0.0257), the dissimilatory nitrate reduction to ammonia (DNRA) pathway (July vs. August: value of *p* = 0.0017; July vs. September: value of *p* = 0.0016), the assimilatory nitrate reduction to ammonia pathway (ANRA) (July vs. August: value of *p* = 0.0007 July vs. September: value of *p* = 0.0001), and the nitrification pathway (July vs. September: value of *p* = 0.0317). There were no temporal variations in the nitrogen fixation or the anammox pathways (Figure 6C). Regarding the phosphorus cycle, the relative read abundance of genes involved in microbial regulation of organic P-mineralization (OPM) was significantly different between July and September (value of *p* = 0.0412). There were no significant temporal variations in the genes involved in inorganic P solubilization or P uptake and transport (IPS-PUT), nor in the genes related to P-starvation response regulation (PSRR; Figure 6D).

**Figure 7:**
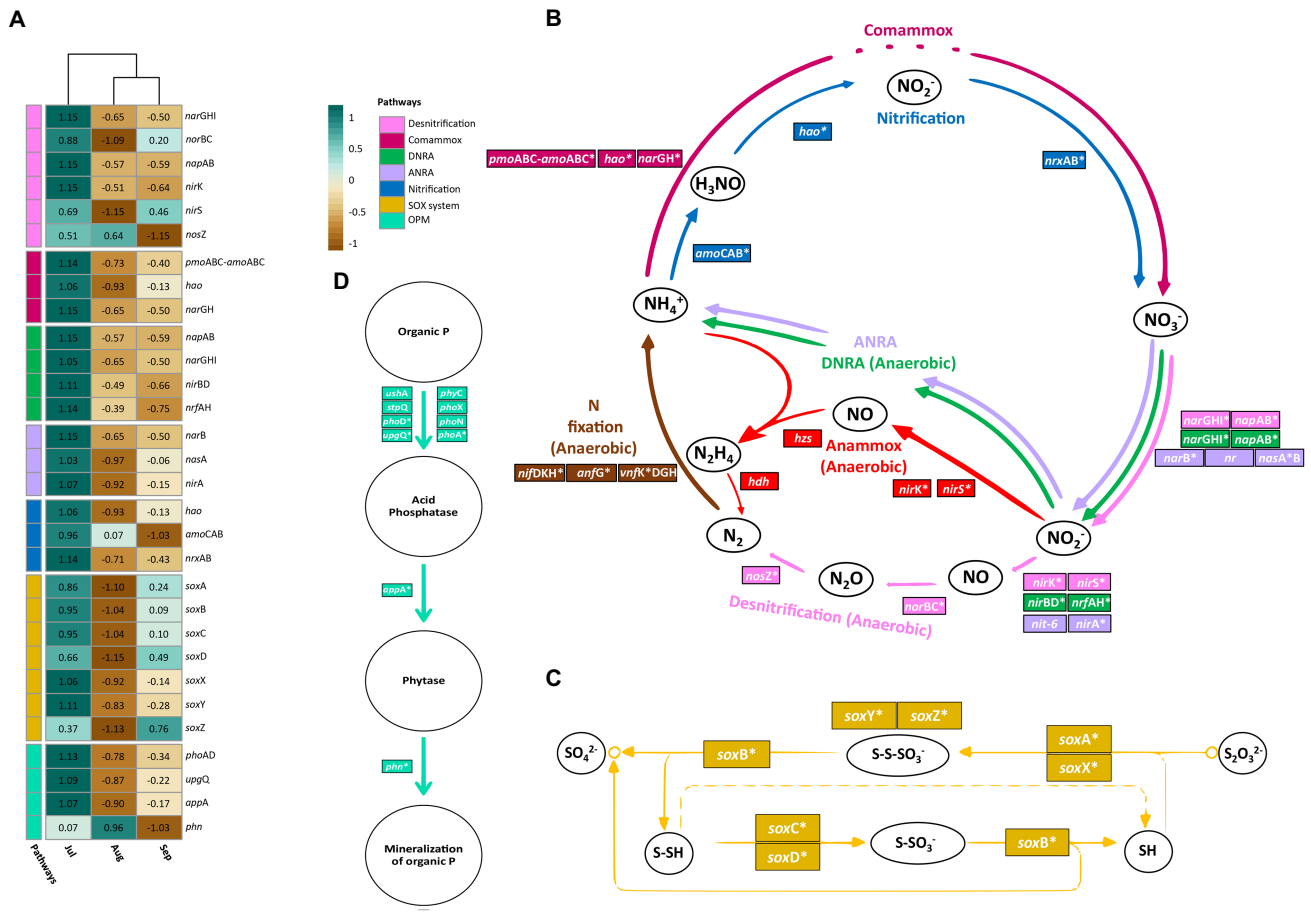
**(A)** Heatmap of significant metabolic pathways and their genes. **(B)** Nitrogen metabolism. **(C)** Sulfur pathway; sulfur oxidation system (SOX system). **(D)** Phosphorus pathway; organic P-mineralization (OPM). Genes with asterisks indicate that they were detected in this study.

The metabolic pathways that were statistically different between sampling month for relative read abundances were further analyzed using an Euclidean distance heatmap and a graphical representation. (Figure 7A). All pathways for the nitrogen cycle are depicted since the majority displayed significant differences between sampling months (Figure 7B). The SOX system and the OPM pathways (Figure 7C) represent sulfur and phosphorus metabolism, respectively (Figure 7D). The pathways involved in central carbon metabolism are not displayed because no temporal variations were detected.

The heatmap reveals that most of the studied genes were overrepresented in July, then declined in August, and then some slightly surged again in September, particularly those of the SOX system pathway (Figure 7D). However, notable exceptions were detected for some genes, such as the *nos*Z gene of the denitrification pathway, which is required to obtain N_2_ from N_2_O, where *nos*Z was more highly represented in July and August and displayed the lowest annotated reads in September. Reads for the *amo*CAB genes, which are necessary to produce hydroxylamine (NH_2_OH) from NH_4_^+^, decreased throughout the months of the study (Figure 7B). The *sox*Z gene, which is required to produce S, SO_3_ and SH in the SOX system pathway of sulfur metabolism, was most abundant in September, followed by July, and August (Figure 7C). Finally, the *phn* gene, which is required for the mineralization of organic phosphorus in the OPM pathway of the phosphorus cycle, was most abundant in August, followed by July and September (Figure 7D).

## Discussion

### The water quality of Lake Cajititlán

Most physicochemical parameters (except for WT and EC) from March to September 2018 were outside of the permissible limits defined by the applicable Mexican regulation and international water quality recommendations (Table 1). This is concerning because the reservoir’s main activity is commercial fishing as well as weekend recreational boat trips, implying an impact on the local economy, i.e., loss of tourism, aesthetic value, fisheries, and seriously affecting the food web dynamics and nutrient balance (de Anda et al., 2019).

The rapid changes in water quality parameters during the wet season have been attributed to runoff caused by heavy rainfall (Díaz-Torres et al., 2021, 2022). Furthermore, significant temporal changes in physicochemical parameters can be observed even during the dry-hot season (March–May; Table 1), indicating that these alterations could be due mainly to climate change impacts on the hydrological cycle of Lake Cajititlán (Inglezakis et al., 2016). Consequently, the physicochemical and biological properties of this body of water are concerning throughout the year. Other research in lakes and estuaries have focused on the influence of climate change on water temperature, nutrient loads, and eutrophication (Kirilova et al., 2011; Chaturvedi et al., 2021). An analysis of the potential effects of climate change in the basin region of Lake Cajititlán would allow for a better understanding of the seasonal dynamics in the reservoir’s physicochemical parameters.

Eutrophication is assessed using the OECD’s trophic status classification, which considers three parameters: TP, chlorophyll-*a*, and secchi disc transparency (Caspers, 1984). According to the findings of this study, Lake Cajititlán is classified as hypereutrophic because the values of these three parameters fell into that category during all 7 months of evaluation (Table 1). This is the worst OECD classification in terms of trophic status and water quality in bodies of water. According to Ram et al. (2014) and Kangur et al. (2005), the main causes for massive fish kills in different bodies of water include natural and anthropogenic hypoxia as well as the proliferation of harmful algae. Accordingly, the presence of phytoplankton biomass as well as fish killed or fish gasping for air on the surface of Lake Cajititlán were observed during the monitoring activities of this study. Moreover, cyanobacteria species that are capable of producing toxins that harm fish were identified through shotgun metagenomics. Five of the seven months studied (Table 1) displayed DO levels below the acceptable limit for aquatic life (5 mg/L; LFD, 2019), which could be driving the mass fish kill episodes in Lake Cajititlán [Smith and Schindler, 2009; Organisation for Economic Co-operatin and Development (OECD)].

### Dynamics and abundance of the microbial communities and their physicochemical influences

There are very few studies on the analysis of microbial communities of a freshwater system, and even fewer a eutrophic state and are being analyzed with shotgun metagenomics. In this have study, approximately 50% of the total reads were classified taxonomically (Table S1). These findings were compared with a similar study, which examined water samples from soda lakes with and without cyanobacterial blooms and using the same metagenomic pipeline as this study. The authors analyzed 24 water samples and obtained about 14 million reads, of which 5,385,139 reads were taxonomically assigned (less than 50%; Andreote et al., 2018). Therefore, it is possible to infer that current genomic databases have limitations and are not representative of water microbiomes. Most of the information added to existing databases comes from pharmaceutical and industrial biotechnology, and human health research efforts, which can mislead genetic annotations of water microbiomes (for example, annotations that are obviously incompatible with water ecosystems), or there is not enough information on water microbial communities to annotate metagenomic studies (Wu et al., 2009; Segata et al., 2012; Huttenhower et al., 2012; Choi et al., 2016). To address the demands in water-related research topics, which have also risen significantly in recent years, it is important to curate databases using genomic data derived from cultured representatives originating from water.

Significant temporal differences were observed in the composition of phytoplankton and bacterial communities between July and September (Supplementary Tables S3, S4). This suggests that the abundance and diversity of these two communities are related, as has been shown in other lakes where changes in environmental factors and phytoplankton communities have influenced the composition of bacterial communities and ecosystem-wide changes (Woodhouse et al., 2015; Ramanan et al., 2016). *Planktothrix agardhii* and *Microcystis aeruginosa* were the most abundant species observed in this study (Figure 3A). These findings are consistent with a study carried out in Lake Cajititlán that analyzed the 16S and 18S rRNA genes to study the phytoplankton communities of this reservoir, in which the genera *Planktothrix* spp. and *Microcystis* spp. were identified. However, the target metagenomic analysis used in that study did not allow for the classification of these phytoplankton groups at the species level (Díaz-Torres et al., 2021). Other studies have found that these two cyanobacterial species are the most dominant in phytoplankton communities, as well as in the harmful algae blooms in other shallow, subtropical, and eutrophic water bodies, such as Lake Cajititlán using other methods (e.g., Malassez counting chamber with a microscope; Kruk et al., 2002; Suda et al., 2002; Bouchamma et al., 2004; Briand et al., 2008; Gągała et al., 2010). This suggests that Lake Cajititlán provides favorable conditions for the development of *P*. *agardhii* and *M. aeruginosa*. Additionally, the genes involved in the production of microcystins (*mcy*-genes) have been investigated, revealing that *Planktothrix* and *Microcystis* predominate in blooms of toxigenic cyanobacteria in freshwater bodies (Dang et al., 2011; Humbert et al., 2013; Ngwa et al., 2014; Harke et al., 2016; Bukowska et al., 2017). Thus, the production of microcystins could be involved in the massive fish kill events that have occurred in Lake Cajititlán. This toxin has already been detected in the water of Lake Cajititlán in a previous study (Díaz-Torres et al., 2021), but toxicological studies regarding bioaccumulation in fish tissues are still needed to confirm this theory.

The most abundant bacterial genera were *Pseudomonas*, *Streptomyces*, and *Flavobacterium* (Figure 4A). This is consistent with other studies that have found these taxa to be among the most abundant genera in freshwater systems, as well as eutrophic and hypereutrophic water bodies (Choi et al., 2005; Michaud et al., 2012; Shao et al., 2021). This suggests that these bacteria thrive in subtropical and eutrophic environments, like Lake Cajititlán. Moreover, they coincide with the most abundant bacterial genera reported by Díaz-Torres et al. (2021) using the 16S rRNA gene, except for *Streptomyces*, which was not even identified as one of the most abundant genera (> 0.01%). Additionally certain genera that were detected herein (*Acidovorax*, *Acinetobacter*, *Aeromonas*, *Agrobacterium*, *Devosia*, *Microbacterium*, *Mycobacterium*, *Mycolicibacterium*, *Nocardioides*, *Rhizobium*, *Rhodococcus,* and *Streptomyces*) were not detected in that previous study, and vice versa (*Aromatoleum*, *Chthoniobacter*, *CL500* and *Cylindrospermopsis*). However, this is expected, as shotgun metagenomic sequencing analysis has potential advantages over meta-barcoding sequencing, such as more genetic information per sample (higher sequencing depth) and the possibility for a better quantitative match (Bell et al., 2021) Furthermore, shotgun metagenomic sequencing covers all genetic information in a sample, so the data may be utilized for metabolic function profiling, metagenomic assembly and binning, antibiotic resistance gene profiling, among other things (Segata et al., 2012; Sunagawa et al., 2013; Laudadio et al., 2019).

NH_4_^+^, TP, TN, ORP, DO, EC, BGA-PC, and turbidity were the most correlated factors with the prevalence of phytoplankton and bacterial communities throughout the research period according to the RDA. The TN and NH_4_^+^ parameters are important water quality factors that influence the distribution of bacterial and phytoplankton communities. These organisms need a substantial quantity of nitrogen for the synthesis of their primary constituents, which include NAD, purines and pyrimidines, amino acids, and amino sugars. Furthermore, both these organisms and archaea are key drivers in the transformation of diverse types of N into chemical forms used by plants (NH_4_^+^ and NO_3_^−^) (Reitzer, 1996; Wang et al., 2018). In Lake Cajititlán, rains have been reported to cause significant fluctuations in nutrient concentrations, which increase during the wet season due to surface runoff containing large amounts of nitrogenous and phosphorous fertilizers (Gradilla-Hernández et al., 2020; Díaz-Torres et al., 2021, 2022). This is consistent with the were higher values of NH_4_^+^ observed in the wet season (June–September; mean = 3.52 mg/L) compared to the dry season (March–May; mean = 2.50 mg/L). In terms of TP, no significant differences were detected. Because microorganisms and plants require P in the form of phosphates, which were not evaluated in this study, the direct influence of this element on bacterial and phytoplankton populations in Lake Cajititlán cannot be correlated (CEDRSSA, 2019; Zheng et al., 2019).

### Analysis of bacterial species reported as pathogenic for fish

The most abundant species during the three months of this study were *Pseudomonas fluorescens*, *Stenotrophomonas maltophilia*, and *Aeromonas veronii* (Figure 5). The most important species in fish pathology is *P. fluorescens*, which is frequently associated with skin problems and fin diseases. Furthermore, infections with this species have been associated with sudden fish kills (Pękala-Safińska, 2018). The ferric uptake regulator gene (*fur*) in the *P. fluorescens* pathogen is essential for optimum infection to occur in fish, as this gene regulates the expression of several proteins (Liu et al., 2015). In this study, this species relative read abundance was greatest in July, which matched with the highest abundance of *fur* gene reads. However, no direct association was observed in August and September (Figure 5, Supplementary Figure S1). *S*. *maltophilia* is commonly isolated from freshwater fish (Juhnke and des Jardin, 1989**;** Dungan et al., 2003), causing lethargy, skin depigmentation, focal hemorrhages, petechiae, and edema in the body cavity, and even fish mortality (Geng et al., 2010a).

*A*. *veronii* has been reported to infect freshwater fish, amphibians, birds, and mammals, causing significant losses in the aquaculture sector and threatening food safety (D’Aloia et al., 1996; Ghenghesh et al., 1999; Zhang et al., 2019). Moreover, *A*. *veronii* can cause infections in humans, particularly the elderly and immunocompromised children, resulting in sepsis, gastroenteritis, and other diseases (Mencacci, 2003; Roberts et al., 2006; Chen et al., 2015). Because this species was the third most abundant in this research, it is imperative that aquatic products from Lake Cajititlán undergo proper food safety inspection before consumption (Figure 5). Previous research shows that the pathogenicity of *A. veronii* in fish is multifactorial. In this study, seven important virulence genes (*aer, act, aha, ser, exu, lip,* and *lux*S) were detected (Supplementary Figure S1; Li et al., 2020). These findings are consistent with an investigation conducted in China, where 203 freshwater fish were collected, and 87 strains of *A*. *veronii* isolated, 50% of which carried at least four or more of the virulence genes evaluated in this work (Li et al., 2020). These results are of great importance for future decision-making with the objective of conserving the ecosystem as well as the food safety of aquatic products from Lake Cajititlán. The infection process of *P. fluorescens, S*. *maltophilia, A. veronii* and other pathogenic microorganisms, together with stressful factors for the fish, could be the cause of the massive kill events that have been reported at this reservoir (Townsend et al., 1992; Glibert et al., 2002; Kangur et al., 2005). However, toxicological and pathological analyses in fish and proteomic analyses are require to determine whether the genes detected in this study are being expressed. Previous studies of Lake Cajititlán evaluated the 16S rRNA gene at the genus level and made theoretical inferences about which species of pathogenic bacteria could be present according to the genera found (Díaz-Torres et al., 2021, 2022). In the present research, use of the shotgun sequencing approach allowed for the characterization of bacteria in this reservoir at the species level, enabling the identification of previously described species, as can be observed in the chord diagram (Figure 5).

### Microbial community biological functions in association to biogeochemical cycles

Significant temporal changes in several pathways of nitrogen, phosphorus, and sulfur metabolism were observed in this study (Figure 6C). Regarding nitrogen metabolism, the *nar*GHI, *nap*AB, and *nir*K genes were the most abundant in the denitrification pathway, which intensified in July (Figure 7A). The first step of the denitrification pathway (NO_3_^−^ to NO_2_^−^) is governed by the first two genes (*nar*GHI and *nap*AB). The second step (NO_2_^−^ to NO) is mainly regulated by *nir*K, because its abundance was higher than *nir*S. The third step (NO to N_2_O) is controlled by *nor*BC and the last step (N_2_O to N_2_) by *nos*Z. However, this last gene was more abundant in August and its lowest abundance was observed in September (Figures 7A,B). This might imply that each stage of this metabolic pathway does not occur sequentially (Howarth et al., 2011). On the other hand, anaerobic conditions with DO values less than 1.7 mg/L are required for nitrogen removal, and thus for conventional biological denitrification (Daigger, 2014). In this study, DO values lower than required were reported at different sites and sampling depths, mainly in April and May (Supplementary Table S9). However, it is well known that the DO in eutrophic water bodies reaches its peak during the day due to the accelerated photosynthetic process carried out by the excessive amount of phytoplankton (monitoring time of this study 8:00-13:00), and it is depleted during the night, attributed to the respiration metabolism, and perhaps as a result, the genes of microorganisms capable of denitrification are present (Smith and Schindler, 2009; Figures 6C, 7A). On the other hand, these results also suggest that facultative anaerobic microorganisms capable of performing complete denitrification, such as *P. aeruginosa, P. fluorescens, P. stutzeri,* and *P. denitrificans*, could carry out the denitrification process at higher concentrations of DO, as demonstrated in a previous study, in which the authors showed that *P. stutzeri* isolated from a wastewater treatment system, can carry out nitrification and denitrification simultaneously in the presence of high levels of oxygen (bottles containing an atmosphere of 92% oxygen; Su et al., 2001; Liou and Madsen, 2008; Arat et al., 2015; Tang et al., 2020). This information coincides with the fact that *Pseudomonas* was the most abundant genus in this study (Figure 6C). In addition, the genes (*nar*GHI, *nor*BC, *nap*AB, *nir*K, *nir*S, *nos*Z) involved in the denitrification process were found annotated in the species *P. flourescens, P. stutzeri,* and *P. aeruginosa* (Supplementary Table S10); this information suggests that it is worth investigating more about facultative anaerobic microorganisms in water ecosystems since it could be very useful for water remediation processes. 

The denitrification process, as well as the anammox, are important in the removal of nitrogen from aquatic ecosystems. However, the anammox pathway showed no temporal variations and was substantially less abundant than the denitrification process (Ward et al., 2009; Bernhard, 2010). Therefore, these results suggest that denitrification is the most important nitrogen removal mechanism in Lake Cajititlán, as previously suggested by Díaz-Torres et al. (2022) using the PICRUSt functional inference approach.

Similar to the denitrification pathway, all genes involved in comammox metabolism were found to be more abundant in July (Figures 6C, 7A). Because this pathway involves the direct conversion of NH_3_ into NO3-by a single *Nitrospira* bacterium, also known as comammox *Nitrospira*, these results correspond to the greatest NH_4_^+^ levels shown in July compared to the other months (Lawson and Lücker, 2018). Comammox bacteria are distinguished from other canonical nitrifiers due to their unique metabolic mechanism (Annavajhala et al., 2018; Palomo et al., 2018). While this genus was not classified as the most abundant bacteria in this study, its presence was identified in the metadata, and it became more abundant in July. Furthermore, the genes involved in the comammox process (*pmo*ABC-*amo*ABC, *hao, nar*GH) were found to be annotated in the phylum Nitrospirae, indicating that this bacterium may be carrying out this metabolism in Lake Cajititlán (Supplementary Table S10).

A greater relative read abundance of DNRA-associated genes was identified throughout the research compared to other nitrogen pathways (Figure 6C), indicating that microbial communities in the water column had a sustained capability to convert NO_3_^−^ to NH_3_. A greater abundance of all the genes (*nar*GHI, *nap*AB, *nir*BD, and *nrf*AH) involved in this metabolic pathway was observed in July, similar to the ANRA pathway. Although the final product of both pathways is the same, ANRA is produced under aerobic conditions when reduced nitrogen is limited, and DNRA occurs when oxygen is limited (anaerobic conditions) and their respective reduced products (NH_4_^+^ and N_2_ + N_2_O, respectively) are produced in greater amounts (Teje et al., 1981; Henson et al., 2017). Based on the OD measurements (Table 1), the genes of the DNRA pathway are more functional in Lake Cajititlán than the genes of ANRA (Figure 6C). This suggests that the massive bloom of phytoplankton in Lake Cajititlán is mostly attributable to the use of nitrogen from chemical fertilizers that reach this reservoir through surface drag, rather than a symbiotic interaction with atmospheric nitrogen-fixing bacteria (Aasfar et al., 2021).

Nitrification is an aerobic nitrogen oxidation pathway that produces NO_3_^−^ from NH_3_ (EPA, 2002). This pathway was the least abundant in the sampled months (Figure 6C), which might be due to the *Nitrospira* species enhancing comamox metabolism more than nitrifying bacteria conducted nitrification; although *Nitrospira* bacteria participate in the nitrification pathway, the whole genetic repertory for ammonia and nitrate oxidation (*pmo*ABC-*amo*ABC), hydroxylamine (*hao*), and nitrate oxidoreductase (*nar*GH) are required for commamox metabolism (Camejo et al., 2017; Palomo et al., 2018). According to genes associated with nitrification metabolism (*hao*, *amo*CAB, *nrx*AB), these were more abundant in July, as were the other nitrogen pathways (Figure 7A). Nitrifying bacteria include species of genera such as *Nitrosomonas*, *Nitrosococcus*, *Nitrobacter*, *Nitrosolobus*, *Nitrosovibrio*, *Nitrospina, Nitrospira*, and *Nitrococcus* (Hagopian and Riley, 1998). In this study, only the *Nitrobacter* and *Nitrospira* genera were detected in the database; for the set of read abundance from these two genera was more abundant in July, followed by September and August, which corresponds with the dynamics of this metabolism (Figure 7A).

All phosphorus metabolic pathways were more abundant in July than in August and September (Ren et al., 2019). The most abundant pathway was organic OPM, followed by IPS-PUT, and PSRR (Figure 6D). Temporal changes were only detected in genes implicated in the OPM pathway (*pho*AD, *upg*Q, *app*A, and *phn*). The increased representation of these genes in July suggests an increase in the P concentration of the water. However, phosphates were not measured in this study, which is how microorganisms and plants utilize P, and no temporal variations were observed for TP (Table 1; Karl, 2000). All genes, except for *phn*, were more abundant in July. The highest abundance of *phn*, which codes for phosphonoacetate hydrolase and is involved in the pathway’s final step, was observed in August (Figures 7A,B). The *phn* gene was more abundant in August, which indicates that there could be a greater organic P mineralization during that month. All the genes involved in the OPM pathway had the lowest abundance in September, which might imply that there was less phosphorus available for the microbial communities in this reservoir.

Regarding sulfur metabolism, the genes associated with the ASR pathway were more abundant than the genes of other metabolic pathways (Figure 6A). Fungi, prokaryotes, and photosynthetic organisms use ASR to convert inorganic SO4_2_^−^ to S_2_^−^, which is then incorporated into amino acid carbon skeletons to produce Cys or homo-Cys (Brunold, 1993). This process occurs in both anaerobic and aerobic conditions and has advantages over the DSR pathway, which only occurs in anaerobic environments (Kushkevych et al., 2020). These findings suggest that the ASR pathway was the most abundant in this study due to the capacity of facultative anaerobic microbes to function both in the presence of DO (i.e., on the surface of Lake Cajititlán), as well as at low DO concentrations (near the sediments of the lake). However, only the SOX system pathway displayed significant temporal variations (Figure 6A). This system, which is present in many sulfur oxidizing bacteria and certain archaea, completely oxidizes reduced sulfur (S) species to SO_4_^2−^ (Quentmeier and Friedrich, 2001; Sauvé et al., 2007; Grabarczyk et al., 2015). The heatmap data shows that all the genes involved in the SOX system pathway were more abundant in July, except for the *sox*Z gene, which was most abundant in September (Figure 7A). Microbial redox reactions of inorganic sulfur compounds are one of the vital processes responsible for the environmental balance of sulfur, mainly sulfur anions. These reactions are mostly mediated by alpha-Proteobacteria sulfur oxidizing (*sox*) genes. *Sox*Z is a sulfur compound chelating protein that binds to *sox*Y and forms a complex with *sox*B, a sulfate thiolesterase, eventually cleaving the sulfur adduct (Bagchi and Ghosh, 2005). As a result, our findings show that the involvement of the sulfur genes is critical since crucial unions are formed to produce the ultimate product, thiosulfate.

Although there were no temporal differences in the carbon cycle pathways, carbohydrate metabolism was the most abundant (Figure 6B). Surface runoff and untreated or partially treated wastewater discharged into Lake Cajititlán by three treatment plants provide an important source of energy for the reservoir’s microbial communities. Furthermore, organic matter pollution increases during the rainy season when wastewater mixes with rain and when treatment plants are over capacity, releasing a mixture of sewage and rainwater into the lake (de Anda et al., 2019; Gradilla-Hernández et al., 2019).

## Conclusion

This research discusses the interactions between abiotic and biotic elements of a subtropical and hypereutrophic lake. Most physicochemical variables (DO, pH, Secchi disk, NH_4_^+^, NO_3_^−^, BGA-PC, TP, and chlorophyll-*a*) were outside of the permissible limits. Likewise, significant temporal variations were observed for most of the physicochemical parameters (DO, pH, WT, Secchi disk, ORP, NH_4_^+^, NO_3_^−^, BGA-PC, chlorophyll-*a*, and EC). These abiotic factors influence the structure of microbial communities, as well as the major nutrient-associated biogeochemical cycles of Lake Cajititlán. The most abundant phytoplankton species identified were *Planktothrix agardhii* and *Microcystis aeruginosa*, both of which are potentially toxic to fish due to their capacity to produce microcystins. *Pseudomonas*, *Streptomyces*, and *Flavobacterium* were the most abundant bacterial genera. *Pseudomonas fluorescens*, *Stenotrophomonas maltophilia*, and *Aeromonas veronii* were the most abundant bacterial species reported as harmful to fish, and some of their genes (*fur*, *lux*S, *aer*, *act*, *aha*, *exu*, *lip*, *ser*) are associated with fish infection. The physicochemical factors most associated with the prevalence of phytoplankton and bacterial communities were NH_4_^+^, TP, TN, ORP, DO, EC, BGA-PC, and turbidity. The genes associated with carbohydrate metabolism, CFPP, DNRA, denitrification, IPS-PUT, OPM, and ASR were found in greater abundance in the microbial communities. Denitrification, comammox, DNRA, ANRA, nitrification, the SOX system, and OPM were the metabolic pathways that displayed significant temporal variations. The genetic evidence presented in this study suggests that the genes for the metabolism of nitrogen, phosphorus, sulfur, and carbon were regulated by anthropogenic nutrient sources reaching Lake Cajititlán, since all metabolic pathways were more abundant in July. This was presumably induced by surface drags produced by rainfall and/or organic matter that enters the lake through the effluents of the WWTPs. Alterations in the biogeochemical cycles have endangered the biodiversity of Lake Cajititlán in recent years. Additional research, such as genome-wide transcriptional profiling of the bacteria directly involved in biogeochemical cycles and associated proteomic analyses, might aid in elucidating the connection between gene transcript abundance, enzyme activity, and its regulation by different nutrient sources. This, in turn, will contribute to a better understanding of the metabolic and ecological niches of microbial communities in aquatic environments.

## Data availability statement

The datasets presented in this study can be found in online repositories. The names of the repository/repositories and accession number(s) can be found at: https://www.ncbi.nlm.nih.gov/sra/PRJNA851822.

## Author contributions

OD-T, MG-H, and CS-G conceived the study and designed the methodology. MG-H, DO-N, and CS-G supervised the manuscript’s preparation. OD-T, JA, and MG-H collected the data. OD-T analyzed the data. MG-H, CS-G, and OL-M were responsible for funding acquisition. All authors contributed to the article and approved the submitted version.

## Funding

Sample sequencing was financed through the 2022 Core Lab Genomics Tec-BASE Seed Fund for Research Projects from Tecnologico de Monterrey.

## Conflict of interest

The authors declare that the research was conducted in the absence of any commercial or financial relationships that could be construed as a potential conflict of interest.

## Publisher’s note

All claims expressed in this article are solely those of the authors and do not necessarily represent those of their affiliated organizations, or those of the publisher, the editors and the reviewers. Any product that may be evaluated in this article, or claim that may be made by its manufacturer, is not guaranteed or endorsed by the publisher.

## Footnotes

^1^ https://www.genome.jp/kegg/ko.html
