# Supplementary material for: Characterizing a subtropical hypereutrophic lake: From physicochemical variables to shotgun metagenomic data
Source: Front Microbiol. 2022 Dec 2;13:1037626. doi: 10.3389/fmicb.2022.1037626 (PMC9755700; doi:10.3389/fmicb.2022.1037626)
Supplement: Supplementary file 1 [file Data_Sheet_1.docx]

Supplementary Tables

**Supplementary Table 1.** Number of reads and contigs obtained from sequencing data analysis at domain level.

| **Domain (30 samples)** | **Total initial reads** | **Total clean reads** | **Total contigs** | **Taxonomic classification of contigs (%)** | **Unclassified contigs (%)** |
| --- | --- | --- | --- | --- | --- |
| Bacteria | 748,721,392 | 731,028,699 | 59,236,568 | 27,457,854 (46.45) | 29,691,987 (50.12) |
| Eukaryota |  |  |  | 1,881,981 (3.17) |  |
| Archaea |  |  |  | 160,851 (0.27) |  |
| Viruses |  |  |  | 43,895 (0.07) |  |

**Supplementary Table 2.** Number of contigs and annotation results by sampling month.

| **Month​** | **n​** | **Total contigs​** | **Taxonomic classification of contigs (%)** | **Total predicted genes** | **EggNOG annotation sequences (%)​** |
| --- | --- | --- | --- | --- | --- |
| July​ | 10​ | 23,077,105​ | 11,534,336 (49.98)​ | 26,937,166 | ​4,897,282 (18.18) |
| August​ | 10​ | 16,657,438​ | 8,615,889​ (51.72)​ | 19,793,200​ | 3,645,666 (18.42)  ​ |
| September​ | 10​ | 18,182,999​ | 9,394,356​ (51.67)​ | ​21,173,907​ | 3,948,617  (18.65) |

**Supplementary Table 3.** Pairwise dissimilarity tests of phytoplankton communities for the different months using ADONIS. The numbers outside the parentheses are “R^2^.” P-values are in parenthesis. Bold values denote statistical significance at the p < 0.05 level.

| **Phytoplankton communities** | **All (P-values)** |
| --- | --- |
| All | 0.13 (0.55) |
| Jul vs. Aug | 0.05 (0.38) |
| Jul vs. Sep | **0.20 (0.05)** |
| Aug vs. Sep | 0.06 (0.21) |

**Supplementary Table 4.** Pairwise dissimilarity tests of the bacterial communities for the different months using ADONIS. The numbers outside the parentheses are “R^2^.” P-values are in parenthesis. Bold values denote statistical significance at the p < 0.05 level.

| **Bacterial communities** | **All (P-values)** |
| --- | --- |
| All | 0.05 (0.62) |
| Jul vs. Aug | 0.03 (0.07) |
| Jul vs. Sep | **0.25 (0.05)** |
| Aug vs. Sep | 0.04 (0.55) |

**Supplementary Table 5.** Redundancy analysis (RDA) of phytoplankton communities. Eigenvalues, proportion explained, and cumulative proportion by 12 RDA axes are shown.

| **Component** | **Eigenvalue** | **Proportion explained** | **Cumulative proportion** |
| --- | --- | --- | --- |
| RD1 | 21.52 | 0.25 | 0.25 |
| RD2 | 13.44 | 0.17 | 0.42 |
| RD3 | 9.30 | 0.13 | 0.55 |
| RD4 | 7.30 | 0.09 | 0.64 |
| RD5 | 5.80 | 0.09 | 0.73 |
| RD6 | 4.87 | 0.07 | 0.8 |
| RD7 | 4.50 | 0.07 | 0.87 |
| RD8 | 4.36 | 0.05 | 0.92 |
| RD9 | 3.71 | 0.03 | 0.95 |
| RD10 | 3.11 | 0.02 | 0.97 |
| RD11 | 2.72 | 0.02 | 0.99 |
| RD12 | 2.22 | 0.01 | 1.00 |

# Supplementary Table 6. Eigenvalues of the physicochemical variables in the RDA of phytoplankton communities. Water temperature (WT), dissolved oxygen (DO), pH, electrical conductivity (EC), ammonium (NH_4_^+^), nitrate (NO_3_^-^), turbidity, oxidation-reduction potential (ORP), blue-green algae (BGA-PC), total nitrogen (TN), total phosphorus (TP).

| Variable | RDA1 | RDA2 | RDA3 | RDA4 |
| --- | --- | --- | --- | --- |
| DO | 0.08 | 0.44 | -0.24 | -0.56 |
| pH | 0.17 | 0.20 | **0.66** | -0.03 |
| WT | 0.14 | -0.17 | 0.11 | **-0.72** |
| Turbidity | 0.06 | 0.19 | **0.66** | 0.07 |
| ORP | **0.41** | 0.11 | **0.74** | 0.04 |
| NH_4_^+^ | 0.07 | **-0.47** | 0.41 | -0.51 |
| NO_3_^-^ | 0.23 | -0.45 | **0.67** | -0.10 |
| BGA-PC | 0.14 | **0.52** | 0.20 | 0.33 |
| Chlorophyll-*a* | 0.25 | 0.39 | -0.08 | -0.05 |
| EC | -0.10 | 0.01 | -0.13 | 0.11 |
| TN | **0.45** | **-0.78** | -0.14 | -0.03 |
| TP | -0.22 | -0.19 | 0.10 | **-0.66** |

**Supplementary Table 7.** Redundancy analysis (RDA) of bacterial composition. Eigenvalues, proportion explained, and cumulative proportion by 12 RDA axes are shown.

| **Component** | **Eigenvalue** | **Proportion explained** | **Cumulative proportion** |
| --- | --- | --- | --- |
| RD1 | 277.01 | 0.39 | 0.39 |
| RD2 | 103.70 | 0.14 | 0.53 |
| RD3 | 62.07 | 0.08 | 0.61 |
| RD4 | 58.13 | 0.08 | 0.69 |
| RD5 | 36.72 | 0.05 | 0.74 |
| RD6 | 33.73 | 0.04 | 0.78 |
| RD7 | 30.12 | 0.04 | 0.82 |
| RD8 | 23.93 | 0.03 | 0.85 |
| RD9 | 23.73 | 0.05 | 0.90 |
| RD10 | 22.46 | 0.03 | 0.93 |
| RD11 | 19.82 | 0.04 | 0.97 |
| RD12 | 18.50 | 0.03 | 1.00 |

**Supplementary Table 8.** Eigenvalues of the physicochemical variables in the RDA of bacterial communities. Water temperature (WT), dissolved oxygen (DO), pH, electrical conductivity (EC), ammonium (NH_4_^+^), nitrate (NO_3_^-^), turbidity, oxidation-reduction potential (ORP), blue-green algae (BGA-PC), total nitrogen (TN), total phosphorus (TP).

| **Variable** | **RDA1** | **RDA2** | **RDA3** | **RDA4** |
| --- | --- | --- | --- | --- |
| DO | **0.30** | -0.23 | **0.26** | **0.75** |
| pH | 0.14 | -0.34 | 0.04 | -0.14 |
| WT | 0.08 | 0.14 | 0.13 | **0.65** |
| Turbidity | 0.11 | -0.27 | 0.02 | -0.14 |
| ORP | 0.26 | -0.15 | -0.02 | -0.41 |
| NH_4_^+^ | -0.13 | 0.15 | 0.17 | -0.06 |
| NO_3_^-^ | 0.05 | 0.06 | -0.12 | -0.40 |
| BGA-PC | **0.30** | **-0.34** | 0.06 | -0.20 |
| Chlorophyll-*a* | 0.25 | **-0.33** | 0.15 | -0.07 |
| EC | -0.16 | 0.01 | -0.11 | -0.21 |
| TN | **0.32** | **0.76** | -0.16 | -0.13 |
| TP | -0.25 | -0.04 | **0.23** | 0.29 |

**Supplementary Table 9.** Water dissolved oxygen concentrations (DO) of Lake Cajititlán by sampling depth from March to September 2018. Bold letters indicate a value ≤ to 1.7 mg/L of DO, which is required for the nitrogen removal process to occur (Daigger, 2014).

| **Sampling point** | **Sampling Depth** | **March** | **April** | **May** | **June** | **July** | **August** | **September** |
| --- | --- | --- | --- | --- | --- | --- | --- | --- |
| CEA-01 | 0.8 | 6.74 | 10.16 | 8.05 | 4.58 | 4.24 | 3.81 | 7.69 |
|  | 1 | 8 | 10.04 |  | 2.57 |  |  |  |
|  | 2 | 4.7 | **1.54** | **1.07** | 2.27 | 2.51 | 2.92 | 6.8 |
| CEA-02 | 0.8 | 15 | 9.36 | 16.8 | 7.75 | 2.58 | 8.17 | 5.74 |
|  | 1.5 | 7.7 | **1.65** | 2.59 | 4.93 | 2.21 | 2.85 | 3.71 |
|  | 3.4 | 5.55 | **0.82** | **0.8** | 3.68 | 2.66 | 2.16 | 3.02 |
| CEA-03 | 0.8 | 10 | 4.01 | 6.76 | 7.16 | 3.04 | 2.64 | 3.07 |
|  | 2 | 5.95 | **1.68** | 2.36 | 4.78 | 2.32 | 2.08 | 2.63 |
|  | 4.2 | 3.1 | **1.17** | **0.85** | 3.22 | 2.09 | 1.83 | 1.95 |
| CEA-04 | 0.8 | 5.44 | **3.83** | 8.61 | 9.93 | 3.14 | 2.63 | 3.69 |
|  | 1.5 | 4.22 | 2.2 | 1.89 | 7.72 | 2.33 | 1.98 | 3.4 |
|  | 3.2 | 2.82 | **1.67** | **0.95** | 4.36 | 2.85 | **1.7** | 3.3 |
| CEA-05 | 0.8 | 4.64 | 6.73 | 8.42 | 12.81 | 2.98 | 4.61 | 3.7 |
|  |  |  | 3.4 | 1.94 | 11.89 | 2.14 | 2.91 | 3.3 |
|  | 2.3 | 2.1 | **1.18** | **0.9** | 11.22 | 2.18 | 1.82 | 4.28 |

**Supplementary Table 10.** Contig annotations of both the affiliated taxonomy and the genes involved in the comammox biogeochemical pathway of the genus *Nitrospira*, as well as the genes involved in denitrification process of the species of *Pseudomonas flourescens*, *Pseudomonas stutzeri*, and *Pseudomonas aeruginosa*.

**Supplementary Figure 1.** Heatmap with virulence factors that play an important role in the development of diseases caused by *Pseudomonas fluorescens* and *Aeromonas veronii* in fish through the study months. Ferric uptake regulator *(fur*), S-ribosylhomocysteinase (*Lux*S), aerolysin (*aer*), cytotoxic enterotoxin (*act*), adhesin (*Aha*), DNases (*exu*), lipase (*lip*), serine protease (*ser*).

1.14

0.59

1.12

1.08

1.00

1.15

1.04

1.12

-0.74

0.56

-0.80

-0.90

-1.00

-0.62

-0.95

-0.81

-0.40

-1.15

-0.33

-0.18

0.00

-0.53

-0.09

-0.31

July

August

September

*fur*

*Lux*S

*aer*

*act*

*Aha*

*exu*

*lip*

*ser*

**Bacteria**

*Aeromonas veronii*

*Pseudomonas fluorescens*

-1

-0.5

0

0.5

1
