# Supplementary figures and images for: Characterizing a subtropical hypereutrophic lake: From physicochemical variables to shotgun metagenomic data
Source: Front Microbiol. 2022 Dec 2;13:1037626. doi: 10.3389/fmicb.2022.1037626 (PMC9755700; doi:10.3389/fmicb.2022.1037626)

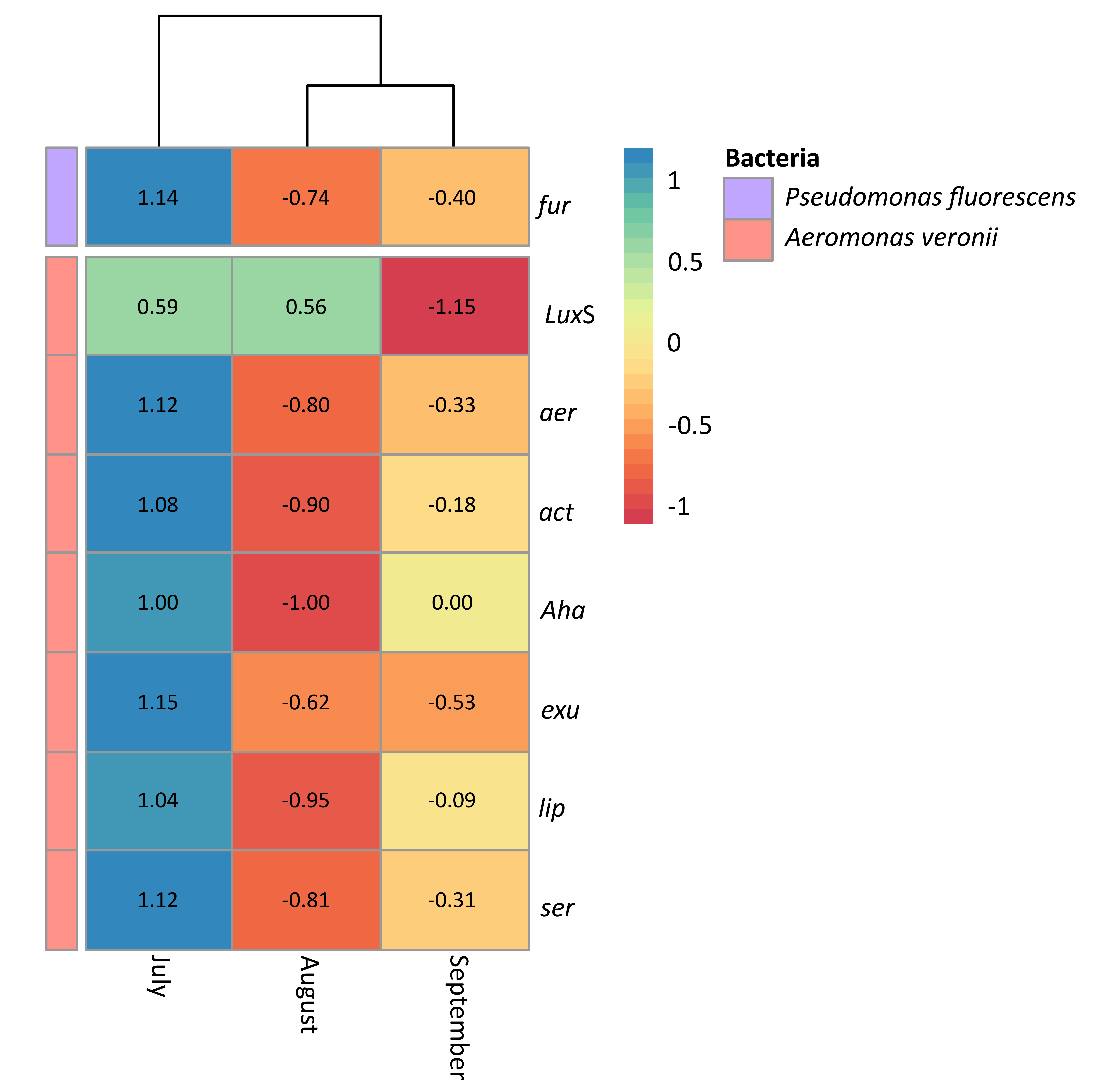

Supplement: Supplementary file 2 [file Image_1.JPEG]
